# 4-Octyl itaconate regulates immune balance by activating Nrf2 and negatively regulating PD-L1 in a mouse model of sepsis

**DOI:** 10.7150/ijbs.74456

**Published:** 2022-10-24

**Authors:** Peng Zhang, Yaxin Wang, Wengchang Yang, Yuping Yin, Chengguo Li, Xianxiong Ma, Liang Shi, Ruidong Li, Kaixiong Tao

**Affiliations:** 1 Department of Gastrointestinal Surgery, Union Hospital, Tongji Medical College, Huazhong University of Science and Technology, Wuhan 430022, China.; 2 Department of Critical Care Medicine, Union Hospital, Tongji Medical College, Huazhong University of Science and Technology, Wuhan 430022, China.

**Keywords:** Itaconate, Sepsis, ROS, Nrf2, PD-L1, Macrophage

## Abstract

**Introduction:** Sepsis is a major global health challenge with high mortality rates and no effective treatment. Recent studies have suggested that sepsis may be associated with immune system dysfunction. Itaconate may exert anti-inflammatory effects via Nrf2 signaling. Although Nrf2 regulates oxidative/exogenous stress responses and inhibits inflammatory responses, the mechanism via which Nrf2 regulates immune checkpoints in sepsis remains unclear.

**Objectives:** This study aimed to investigate the role of the Nrf2 signaling pathway in sepsis immunosuppression injury by exploring Nrf2 target genes in inflammatory macrophages in a mouse model of sepsis.

**Methods:** We evaluated the effects of 4-octyl itaconate (OI) on pro-inflammatory and anti-inflammatory cytokines in a mouse model of sepsis and RAW264.7 cells. In addition, we investigated if OI could inhibit LPS-induced oxidative stress by activating Nrf2 signaling *in vitro* and *in vivo*.

**Results:** OI reduced the release of pro-inflammatory cytokines and increased the release of anti-inflammatory cytokines, thereby inhibiting inflammation. OI increased glutathione synthase (GSS) expression by activating the Nrf2 signaling pathway to promote GSH synthesis, thus, inhibiting oxidative stress. OI inhibited the early release of inflammatory and oxidative stress-related factors to reduce tissue and organ injury in mice with sepsis, while Nrf2 interfered with PD-L1 induction and inhibited PD-L1 expression at an advanced stage to reduce the occurrence of sepsis immunosuppression.

**Conclusions:** This study indicates that Nrf2 is a novel negative regulator of PD-L1 that functions at immune checkpoints and suggests an underlying mechanism for the anti-inflammatory process mediated by Nrf2.

## Introduction

Sepsis is a major global health challenge with high mortality rates. Due to the limited effective treatments available, prompt diagnosis, fluid resuscitation, and immediate treatment with appropriate antibiotics are essential to mitigate associated symptoms and reduce sepsis exacerbation 1-4. However, antibiotic resistance and related side effects seriously affect the treatment effectiveness 5-8.

Recent studies have proposed that sepsis may be associated with immune system dysfunction 9-12. The immune dysfunction includes not only excessive inflammation but also immunosuppression. In the past, immune deregulation of sepsis was considered immune paralysis, followed by an early stage of hyperinflammatory response. However, this view has been considered outdated. Currently, the immune disorder in sepsis is defined as immune overactivation that coexists with immune paralysis even in the early phase of sepsis 13. Immune dysfunction may be caused by the excessive production of endogenous anti-inflammatory mediators. Hence, it is essential to elucidate the role of immune response and function in sepsis to develop effective treatment strategies 14-16. Immunotherapies for sepsis primarily involve the reduction of human leukocyte antigen DR-correlation (HLA-DR), lymphocyte rearrangement (from Th1/M1 immune cell phenotype to Th2/M2 phenotype), induced programmed cell death or apoptosis, and the increased expression of anti-inflammatory mediators (prostaglandin E, interleukin (IL)-10, steroid hormone) 17-19. In particular, the co-inhibition of programmed death 1 (PD-1) and its ligand (PD-L1) help to induce immunosuppression in patients with sepsis 20-22.

PD-L1 is an important immunosuppressive molecule primarily expressed on the surface of antigen-presenting cells such as macrophages and dendritic cells and can functionally combine with PD-1 and PD-2 in the B7 protein family 10, 23, 24. Upon binding a ligand, the PD-1 immunoreceptor tyrosine-based motif region tyrosine is phosphorylated, which induces immune suppression effects by transducing negative signals 25, 26. The PD-1/PD-L1 signaling pathway plays an important role in tumor immunity, infectious diseases, autoimmune diseases, and drug resistance mechanisms. There are many reports that PD-L1 expression is enhanced in different cell types in sepsis. The expression of PD-L1 in splenic capillary endothelial cells in sepsis caused by splenic infarction was higher than in patients with cerebral infarction whose spleen death or trauma required emergency splenectomy 27. PD-1 expression is upregulated on T lymphocytes and PD-L1 on monocytes in patients with septic shock 28. PD-L1 expression in monocytes was associated with 28-day mortality in sepsis 29. These studies suggest that abnormal activation of the PD-1/PD-L1 pathway is the main cause of immune paralysis in patients with sepsis. In addition, the PD-1/PD-L1 pathway may be relevant in sepsis pathogenesis because sepsis also induced immune paralysis in a mouse model of sepsis. Huang *et al.* have shown improved survival of PD-1 knockout mice in sepsis caused by bacterial peritonitis induced by cecal ligation puncture-induced mouse model surgery 30. In a follow-up study of this study, PD-1 treatment of bacterial and fungal or PD-L1 antibodies in septic animal models improved overall survival 31-33. Thus, sepsis could be effectively controlled by inhibiting the expression of immune checkpoint molecules, such as PD-1/PD-L1 and CTLA-4.

During sepsis, most reactive oxygen species (ROS) are derived from phagocytes, among which monocytes/macrophages are the most important 34, 35. When stimulated by a pathogen, phagocytes synthesize and secrete numerous pro-inflammatory cytokines, causing a respiratory burst, which ultimately induces the release of ROS, thereby increasing oxidative stress 9, 36, 37. The most important intracellular antioxidant is glutathione (GSH), which is widely distributed in various tissues and organs. The -SH group of GSH has strong reducibility and can be inactivated by binding to free radicals; therefore, GSH can help cells and tissues to resist oxidative stress damage by scavenging cellular ROS 34, 37, 38.

Nuclear factor erythroid 2-related factor 2 (Nrf2) is an important transcription factor that primarily mediates the cellular antioxidant stress response. Numerous studies have suggested that the Nrf2-Keap1-antioxidant response element (ARE) signaling pathway plays important roles in the anti-tumor activity, drug resistance, neuroprotection, and regulation of inflammation. For instance, Nrf2 activation in myeloid cells has been shown to reduce inflammation. Hence, the Nrf2 inducer, Tecfidera, is used to treat multiple sclerosis due to its anti-inflammatory properties 39-42. In addition, Nrf2 deficiency has been shown to aggravate inflammation in mouse models of ischemia-reperfusion injury, emphysema, autoimmune disorders, and sepsis. Therefore, the complex regulatory mechanism underlying Nrf2 activity is an attractive drug target 43-45.

Under normal conditions, Nrf2 binds to the E3 ubiquitin ligase adapter protein, Keap1, and is subsequently degraded. Conversely, oxidative/exogenous stress blocks Nrf2 degradation, leading to its rapid accumulation and translocation into the nucleus, forming a heterodimer with a small Maf protein 43, 45-47 and regulating the transcription of target genes by binding to their regulatory regions. However, the mechanism underlying its anti-inflammatory effects remains unclear 9, 42. Since Nrf2 upregulates several antioxidant genes, Nrf2 restrains inflammation and may therefore involve ROS elimination. Recent reports have shown that ROS elimination can inhibit inflammation in Nrf2-deficient mice. However, Nrf2 also regulates the expression of macrophage-specific genes associated with the anti-oxidative stress response, such as those encoding MARCO, a receptor required by bacterial phagocytes, and CD36, an oxidative LDL receptor associated with atherosclerosis. Thus, Nrf2 may also act as an anti-inflammatory regulator in a ROS-independent manner 4, 48-50.

Itaconate, a product of citrate distillation, is a derivate of the tricarboxylic acid (TCA) cycle and has been shown to exert anti-inflammatory effects in systemic lupus erythematosus, acute colitis ansd liver ischemia/reperfusion injury 51. Mills *et al.* demonstrated that itaconate is essential for Nrf2 activation in mouse macrophages and can thereby limit inflammation and regulate type I interferon 52-55. In addition, itaconate can activate Nrf2 to protect against lipopolysaccharide (LPS)-induced lethality and reduce cytokine production 56, 57. To determine whether the Nrf2 signaling pathway is involved in sepsis immunosuppression injury, we investigated if 4-octyl itaconate (OI), an itaconate derivative, could inhibit LPS-induced oxidative stress by activating Nrf2 signaling in inflammatory RAW264.7 macrophages and in a mouse model of sepsis. Our study provides further insights into the role of Nrf2 in regulating inflammatory immune status and provides important data for future studies of mechanisms and drug development for sepsis.

## Materials and Methods

### Reagents

OI, N-acetyl-L-cysteine (NAC), tert-butylhydroquinone (TBHQ), and ML385 were purchased from Med Chem Express (USA). Kits used to detect alanine transaminase (ALT) and aspartate transaminase (AST) levels were purchased from the Nanjing Jiancheng Institute of Biotechnology (Nanjing, China). 2,7-Dichlorofluorescein diacetate (DCFH-DA) and reduced glutathione/oxidized glutathione (GSH/GSSG) were obtained from Beyotime Biotechnology (Shanghai, China). Mouse IL-10, IL-1β, interferon (IFN)-γ, and tumor necrosis factor (TNF)-α enzyme-linked immunosorbent assay (ELISA) kits were obtained from Dakewe Bioengineering (Shenzhen, China). Anti-PD-L1, anti-iNOS, Anti-Arg-1, anti-IL-6, anti-IL-1β, Anti-Nrf2 antibodies were purchased from Cell Signaling Technology (Beverly, MA, USA). Anti-HO-1 was purchased from Abcam (USA). Anti-TNF-α, anti-NQO-1, anti-GSS and anti-glyceraldehyde 3-phosphate dehydrogenase (GAPDH) antibodies were purchased from Proteintech (Wuhan, China). Anti-CD86, anti-F4/80, anti-CD206, and anti-CD274 antibodies were purchased from Biolegend (USA). ChIP-grade Protein G Magnetic Beads (#9006) were purchased from Cell Signaling Technology (Beverly, MA, USA). All other chemicals were of the highest commercial grade.

### Animal experiments

Adult male C57bl/6 mice (8-week-old; 20-25 g) were purchased from Beijing Huafukang Biotechnology (Beijing, China). All animal experiments were approved by the Animal Care and Use Committee of Tongji Medical College of Huazhong University of Science and Technology. Mouse models of sepsis were established by cecum ligation and puncture (CLP). The mice were randomly divided into four groups (*n* = 12 per group): (1) Control group, intraperitoneal injection with 400 μL normal saline; (2) CLP group, surgery followed by intraperitoneal injection with 400 µL normal saline; (3) CLP + OI (25 mg/kg) group, surgery followed by intraperitoneal injection with 400 µL OI solution; (4) CLP + OI (50 mg/kg) group, surgery followed by intraperitoneal injection with 400 µL OI solution. Survival rates were evaluated at 12, 24, 36, 48, 72, and 96 h following the indicated treatments.

Alternatively, 24 h post-surgery, mice were washed to collect intraperitoneal cells, and blood samples were collected from the heart. Mice were then sacrificed to collect liver and lung tissues. Histological changes, the ratio of neutrophils and monocytes/macrophages, and changes in inflammatory cytokines (TNF-α, IL-1β, IFN-γ, and IL-10) were observed. OI was administered for 24 h per treatment.

Another set of mice was randomly divided into four groups (*n* = 12 per group): (1) Control operated group; (2) CLP group; (3) anti-PD-1 (200 ng/kg) group, surgery followed by intraperitoneal injection with 400 µL anti-PD-1 solution; (4) anti-PD-L1 (200 ng/kg) group, surgery followed by intraperitoneal injection with 400 µL anti-PD-L1 solution. Anti-PD-1 and anti-PD-L1 were injected intraperitoneally for the first time at 2 hours postoperatively and then at 2 and 4 days postoperatively. The survival rates in each group were evaluated at 1, 2, 3, 4, 5, and 6 days post-surgery. PD-L1 and PD-1 were injected every other day.

### Cell culture and treatment

Murine macrophage-like RAW 264.7 cells were kindly provided by the Cell Bank of the Chinese Academy of Science (Shanghai, China) and were maintained in high glucose Dulbecco's modified Eagle's medium (DMEM; HyClone) supplemented with 10 % fetal bovine serum (ScienCell, Carlsbad, CA, USA) at 37 °C under a humidified 5% CO_2_ atmosphere. During the experiments, the RAW264.7 cells were treated with LPS (1 μg/mL), OI (62.5 μM), or OI (125 μM). The cells were also pretreated with NAC (4 mM), a specific ROS inhibitor. The Nrf2 signaling pathway-specific inhibitor ML385 (5 µM) and Nrf2-specific agonist TBHQ (10 µM) were used to measure oxidative stress indicators and determine protein expression. We strictly controlled the time of the cell experiment. When the cells reached our predetermined density, LPS was used to treat the cells, and OI and other drugs were added to treat the cells 30 minutes later. The next experiment was carried out after incubation for 12 h in a warm box. However, for RNA-seq and Chip-seq experiments, we incubated the cells in a warm box for 24 h and then conducted the sequencing experiments.

### Peritoneal macrophages and cell culture

Male C57 mice were randomly divided into three groups (*n* = 8 per group): Control, shNC, and shNrf2 (The lentivirus titer was 1×10^8^ PFU/ml, and mice were injected 100 ul of concentrated virus through the tail vein and re-injected 7 days apart. Experimental verification was performed 14 days later). Mouse peritoneal macrophages were extracted after two successive injections. Mice were sacrificed by cervical dislocation, and their skin was disinfected with 75% alcohol. After the peritoneum had been exposed, the peritoneal wall was swabbed with 70% alcohol, and the abdominal cavity was injected with 10 mL of sterile PBS using a syringe. The peritoneal wall was rubbed from both sides to ensure that the fluid flowed fully into the abdominal cavity. The PBS was then carefully extracted, injected into a 15-mL centrifuge tube, and centrifuged at 1,000 rpm at 4 °C for 10 min. After the supernatant had been discarded, the sample was centrifuged twice more and resuspended in a complete culture medium. Blood cells were counted under a microscope and inoculated (2 mL/well) at a density of 8.0 × 10^4^ to 1.0 × 10^5^ cells/mL into 6-well plates pre-coated with RPMI 1640 containing 10% fetal bovine serum. Mouse macrophages were cultured for 24 h after fusion and treated with LPS (1 μg/mL) and OI (125 µM). The GV248 lentiviral vector expressing two different short hairpin RNAs (shRNA) targeting mouse. shNC:TTCTCCGAACGTGTCACGT. shNrf2:GCTCGCATTGATCCGAGATAT.

### Histologic analysis of liver and lung tissues

Liver and lung samples were fixed with 4% PFA, embedded in paraffin, and stained with hematoxylin and eosin (H&E). Necrosis was assessed using liver and lung injury scores according to standard morphological criteria.

### Determination of liver enzyme levels

Serum was acquired from blood samples centrifuged at 800 × rpm for 15 min. AST and ALT activities were measured using a microplate reader with assay kits according to the manufacturer's instructions.

### Cytokine quantification by ELISA

IL-6, IL-1β, TNF-α, IL-10, and IFN-γ concentrations in mouse serum samples and the culture supernatant of RAW264.7 macrophages were determined using the respective ELISA kits according to the manufacturer's instructions.

### Evaluation of intracellular antioxidant capacity

RAW264.7 cells were inoculated into 6-well plates, cultured to 40 % confluence, and then treated according to the group descriptions above. After 12 h, cells were collected for experiments, and the probe was diluted with a serum-free culture medium according to the manufacturer's instructions. The RAW264.7 cell culture solution was removed, and 1 mL of the diluted probe was added before incubation at 37 °C for 20 min while shaken at a uniform speed. The cells were carefully washed three times with serum-free cell culture medium to remove extracellular probes and then subjected to flow cytometry using a FACS Canto II flow cytometer (BD Biosciences, San Jose, CA, USA). The total intracellular GSH levels and GSH/GSSG activity in RAW264.7 cells were measured using kits according to the manufacturer's instructions.

### Real-time quantitative polymerase chain reaction (qPCR)

RAW264.7 cells were seeded in 6-well plates at a density of 2 × 105 cells/well and treated for 12 h. cDNA was obtained by reverse transcription using Prime Script TMRT Master Mix (RR036A). The PCR solution was prepared on ice using a Step One Plus real-time PCR System instrument with TB Green TMP remix ExTaq™ (RR420A) as follows: Pre-denaturation at 95 °C for 30 s followed by 40 cycles of 95 ℃ for 5 s and 60 °C for 30 s. Gene amplification specificity was determined by drawing a dissolution curve based on the experimental results. Relative gene expression was calculated using the 2-ΔΔ CT method. qPCR primers included the following B7-H1(1):F: GCTCCAAAGGACTTGTACGTG,R:TGATCTGAAGGGCAGCATTTC.B7-H1(2):F:GTCAATGCCCCATACCGCAA,R:GGCCTGACATATTAGTTCATGCT.Nrf2:F:TCTTGGAGTAAGTCGAGAAGTGT,R:GTTGAAACTGAGCGAAAAAGGC.GAPDH:F:TCTTGGAGTAAGTCGAGAAGTGT,R:TGTAGACCATGTAGTTGAGGTCA.

### Immunofluorescence staining

RAW264.7 cells and peritoneal macrophages were processed accordingly, washed three times with TBS for 5 min each, and incubated in 10 % donkey serum for 30 min. Corresponding primary antibodies were added and incubated at 4 °C overnight. For double staining, cells were reheated for 15 min, washed three times with TBS for 5 min each, and incubated with corresponding secondary antibodies (1:100) at room temperature for 1 h. After being washed three more times with TBS for 5 min each at 4 °C and incubated with 4′,6-diamino-2-phenylindole (DAPI, 1:500) for 10 min to stain the nuclei, the cells were washed three times with TBS for 5 min each and examined using fluorescence microscopy with an anti-fluorescent quenching agent.

### Western blot analysis

Protein samples (10 µg) were carefully added to each well, and 3 µL of pre-stained protein maker was added to each side based on the concentration of the target protein. Electrophoresis was performed at a constant 120 V until the lower edge of the gel was reached, and then a gel of appropriate width was cut and carefully transferred onto the film clip. Electrophoresis was performed at a constant current of 240 mA for 90 min. The proteins were then transferred to a PVDF membrane which was placed in a 5% skim milk powder solution and sealed under a shaker for 1 h. After washing five times with double steaming water, the membrane was incubated with primary antibodies at 4 °C overnight, washed three times with TBST solution for 10 min each, and then incubated with secondary antibodies at room temperature for 1 h. After a further three washes with TBST solution for 10 min each, ECL Solutions A and B were added dropwise, and the target strip was detected using a gel imager.

### Lentiviral infection

RAW264.7 cells grown in the logarithmic phase were resuspended and seeded onto six-well plates at 2 × 10^6^ cells/well density. When the cells reached 30-50 % confluence, the medium was carefully removed, and 960 μL of complete fresh medium, 40 µL of infection enhancers, and virus (multiplicity of infection = 50) were added in this order. After gentle horizontal shaking for 12 h, the culture medium containing the virus was completely removed, and 2 mL of fresh complete culture medium was added to each well. After 12-24 h of culture, puromycin (final concentration: 4 g/mL) was added to each well three times to screen stably-infected cells. Infection efficiency was observed after 12 h using fluorescence microscopy, and follow-up tests were conducted. Nrf2 and KEAP1 shRNA lentiviruses were synthesized and packaged by Shanghai Gikekian Chemical Technology. The GV248 lentiviral vector expressing two different short hairpin RNAs (shRNA) targeting mouse. shNC: TTCTCCGAACGTGTCACGT. shNrf2: GCTCGCATTGATCCGAGATAT; shKEAP-1: GTGCATCGACTGGGTCAAATA.

### Flow cytometry

Cells were inoculated in a 6-well plate and treated according to the group descriptions when they reached 40 % confluence. After 12 h, the cells were collected according to the cell passage experiment method, and the cell density was adjusted to approximately 1 × 10^6^ cells/mL. The cell suspension was carefully transferred into a flow tube, centrifuged at 1,200 rpm for 5 min, and the supernatant carefully discarded. The cells were gently resuspended in 2 mL pre-cooled PBS, blown with a 1 mL pipette gun, centrifuged at 1,800 rpm for 5 min, and the supernatant was carefully absorbed. This step was repeated three times, and the supernatant was removed completely. After the cells had been resuspended in 100 µL of pre-cooled PBS and blown evenly with a 100-µL nozzle, the single-cell suspension was incubated with corresponding antibodies at 4 °C in the dark for 30 min and then subjected to flow cytometry using a FACS Canto II flow cytometer (BD Biosciences).

### siRNA

During the logarithmic phase, cells were resuspended in a complete culture medium, inoculated into a 6-well plate at a density of 2 × 10^6^ cells/well, and transfected with siRNA when they reached 30-50 % confluence. The transfection agents (10 pM siRNA, 50 Opti-MEM, and 2 lipofectamine RNAi MAX) were mixed, left to stand at room temperature for 5 min, mixed, and allowed to stand at room temperature for an additional 20 min. After the medium had been removed from the 6-well plate, the mixture and 900 µL of complete medium without double antibodies were added, mixed, and cultured in the incubator. After 6 h, the supernatant was removed from the plate, and the cells were cultured with 2 mL of complete medium for 24 h. siRNAs were used to silence the expression of Nrf2. Nrf2 siRNA#1: TGTTGGATTCAAGGCTGGTGTTAAA. Nrf2siRNA#2: GATTCAAGGCTGGTGTTAAAGATTA. SiNC TTCTCCGAACGTGTCACGT.

### RNA-sequencing (RNA-seq)

RAW264.7 cells were inoculated into 6-well plates and treated according to the group descriptions when they reached 40 % confluency. After 24 h, the complete culture medium was removed, the cells were washed twice with PBS, and 1 mL RNAiso Plus was added to each well. The samples were stored at -80 °C prior to sequencing and analysis using BGI.

### ChIP-sequencing (ChIP-seq)

RAW264.7 cells were inoculated in 15-cm petri dishes and treated according to the group descriptions when they reached 40% confluency. After 24 h, the cells were collected and processed according to the manufacturer's instructions to obtain purified DNA. The library for high-throughput DNA sequencing libraries was prepared using the VAHTS Universal DNA library preparation kit for Illumina V3 (Cat No: ND607 Vazyme). A range of 200-500 BPS libraries was enriched, quantified, and sequenced using a Novaseq sequencer (Illumina).

### Luciferase reporter assay

One day before transfection, HEK293T cells were inoculated into 96-well plates at 2 × 10^4^ cells/well density and incubated at 5% CO_2_ and 37 °C. The transfection system was prepared as follows: Solutions A and B were mixed carefully and allowed to stand for 20 min at room temperature; 20 µL transfection compound was carefully added to each well and incubated. Luciferase activity was measured using a Dual-Luciferase Reporter Assay System (E1910; Promega). The ratio of luciferase activity was calculated as Luciferase ratio = F/R, where F represents Firefly luciferase, and R represents Renilla luciferase.

### Statistical analyses

Results are presented as the mean ± standard deviation. Statistical analyses were performed using one-way analysis of variance (ANOVA) followed by *t-*tests to determine significant differences between the two groups. All statistical analyses were performed using SPSS software (v.20.0; SPSS, Chicago, IL, USA). *P* values of < 0.05 were considered significant.

## Results

### OI exerts a protective effect against sepsis in mice

**Figure 1:**
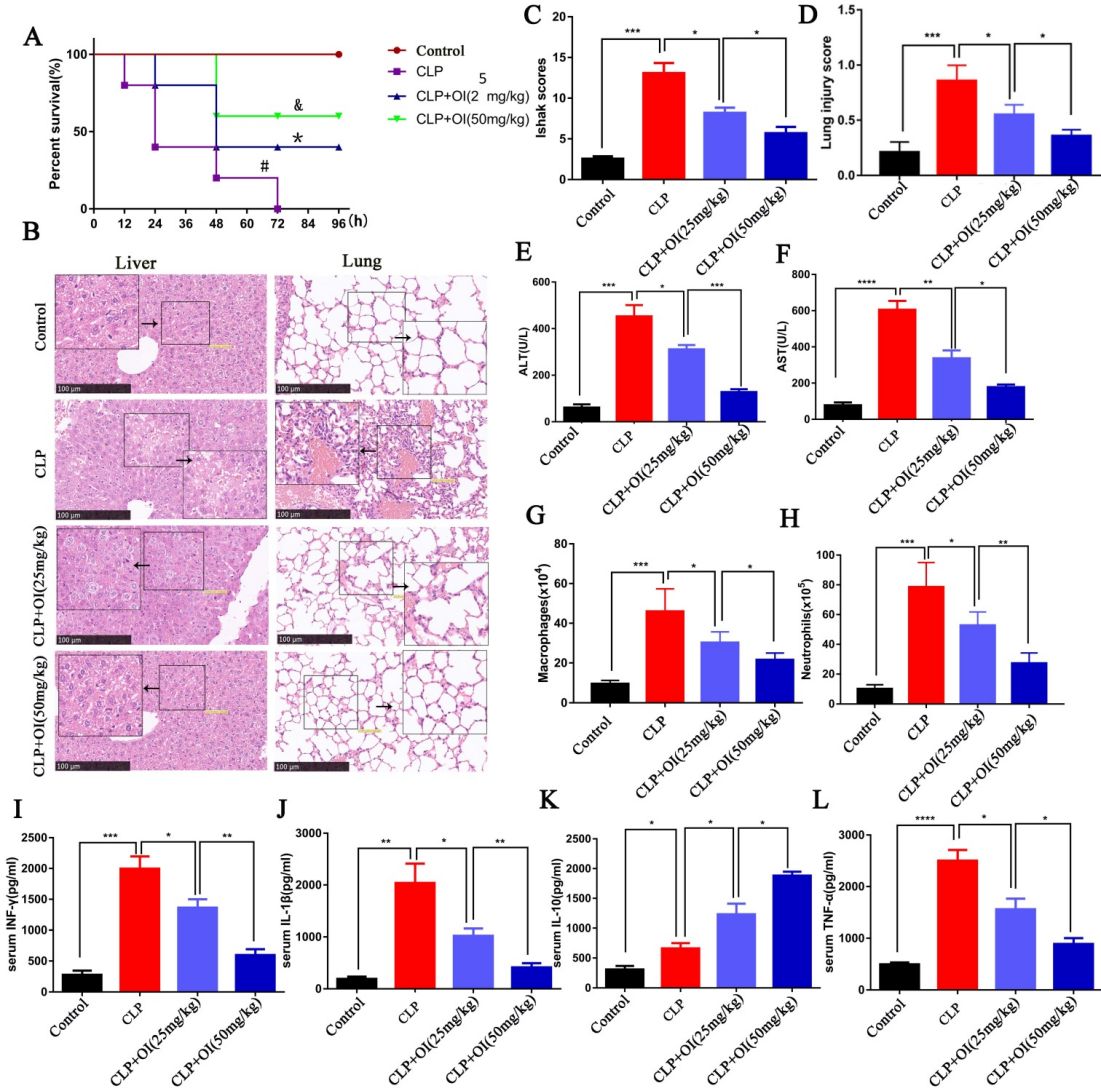
** OI exerts a protective effect against sepsis in mice. (A)** OI reduces mortality in mice with sepsis. ^#^*P* < 0.05 vs. Control; *P < 0.05 vs. CLP; ^&^*P* < 0.05 vs CLP+OI (25 mg/kg). **(B and C)** Pathological injury and score of mouse livers assessed by H&E staining; magnification, 40×. **(E and F)** Changes in ALT and AST liver function in mice. **(B and D)** Pulmonary lesions and scores of mice assessed using H&E staining. **(G)** Changes in mouse peritoneal macrophages. **(H)** Changes in neutrophils from the abdominal cavity of mice. **(I-L)** Pro-inflammatory (IFN-γ, IL-1β, TNF-α) and anti-inflammatory (IL-10) cytokine levels in mouse blood, (n=12). All data are pooled from two independent experiments. Data represent the mean ± SD. ****P < 0.001, ***P < 0.001, **P < 0.01, *P < 0.05.

First, we evaluated the protective effect of OI on mice using the CLP model of sepsis. Mice in the control group had a survival rate of 100% within the observed time range (0-96 h), whereas the survival rates of the CLP group were 83.3 % at 12 h, 66.7% at 24 h, 25% at 48 h, and 8.3% at 72-96 h. In the CLP + OI (25 mg/kg) group, the survival rate was 100% at 12 h, 83.3 % at 24 h, and 66.7 % at 48-96 h, while the CLP + OI (50 mg/kg) group had survival rates of 100% at 0-48 h and 75.0 % at 48-96 h. Therefore, OI significantly reduced the mortality of septic mice in a dose-dependent manner (Fig. 1A).

The liver cells in the control group appeared normal and lacked inflammatory cell infiltration, whereas those from the CLP group showed obvious bubble-like degeneration and steatosis, nuclear shrinkage, cytoplasmic shrinkage, and nucleo-cytoplasmic separation, alongside several infiltrated inflammatory cells. In the CLP + OI (25 mg/kg) group, a small number of hepatocytes showed nuclear atrophy, cytoplasmic atrophy, and inflammatory cell infiltration. However, these lesions were milder than those in the CLP group. Moreover, CLP + OI (50 mg/kg) liver cells were surrounded by some inflammatory cells but did not display noticeable pathological changes (Fig. 1B). The liver tissue damage scores were consistent with the pathological changes indicated by H&E staining (Fig. 1C). Finally, the liver function (ALT and AST) was significantly impaired in the CLP group with ALT and AST levels being significantly increased. Together, these findings suggest that OI can significantly reduce CLP-induced liver function injury in a dose-dependent manner (Fig. 1E-F).

Next, we evaluated the protective effect of OI on lung injury in septic mice. The control group had normal-shaped alveoli with a complete structure and no inflammatory cell infiltration. In contrast, the alveoli of the CLP group mice were damaged, with a significantly thickened alveolar septum and considerable inflammatory cell infiltration. Alveolar destruction and inflammatory cell infiltration were significantly reduced in the CLP + OI (25 mg/kg) group compared with the CLP group and in the CLP + OI (50 mg/kg) group compared to the CLP + OI (25 mg/kg) group (Fig. 1B). Moreover, the lung tissue injury scores were consistent with the pathological changes observed using H&E staining (Fig. 1D).

We also observed key inflammatory cell changes in the abdominal lavage fluid. The number of neutrophils and monocytes/macrophages was significantly higher in the CLP group than in the control group. However, it decreased in the CLP + OI group in a dose-dependent manner (Fig. 1G-H).

Inflammatory cytokine storms are a key cause of deterioration in patients with sepsis. To determine the anti-inflammatory effects of OI, we examined the effect of OI on the release of inflammatory factors in mice with CLP-induced sepsis. No significant changes in cytokine release were observed in the control group; however, the release of the pro-inflammatory cytokines TNF-α, IL-1β, and IFN-γ were significantly higher in the CLP group, as well as that of the anti-inflammatory cytokine, IL-10. Consistently, TNF-α, IL-1β, and IFN-γ levels were significantly lower in the CLP + OI groups than in the CLP group, whereas IL-10 levels were higher in the CLP + OI groups than in the CLP group. These anti-inflammatory effects were positively correlated with OI dose (Fig. 1I-L).

### OI reduced RAW264.7 M1 polarization, increased M2 polarization, and inhibited LPS-induced oxidative stress

**Figure 2:**
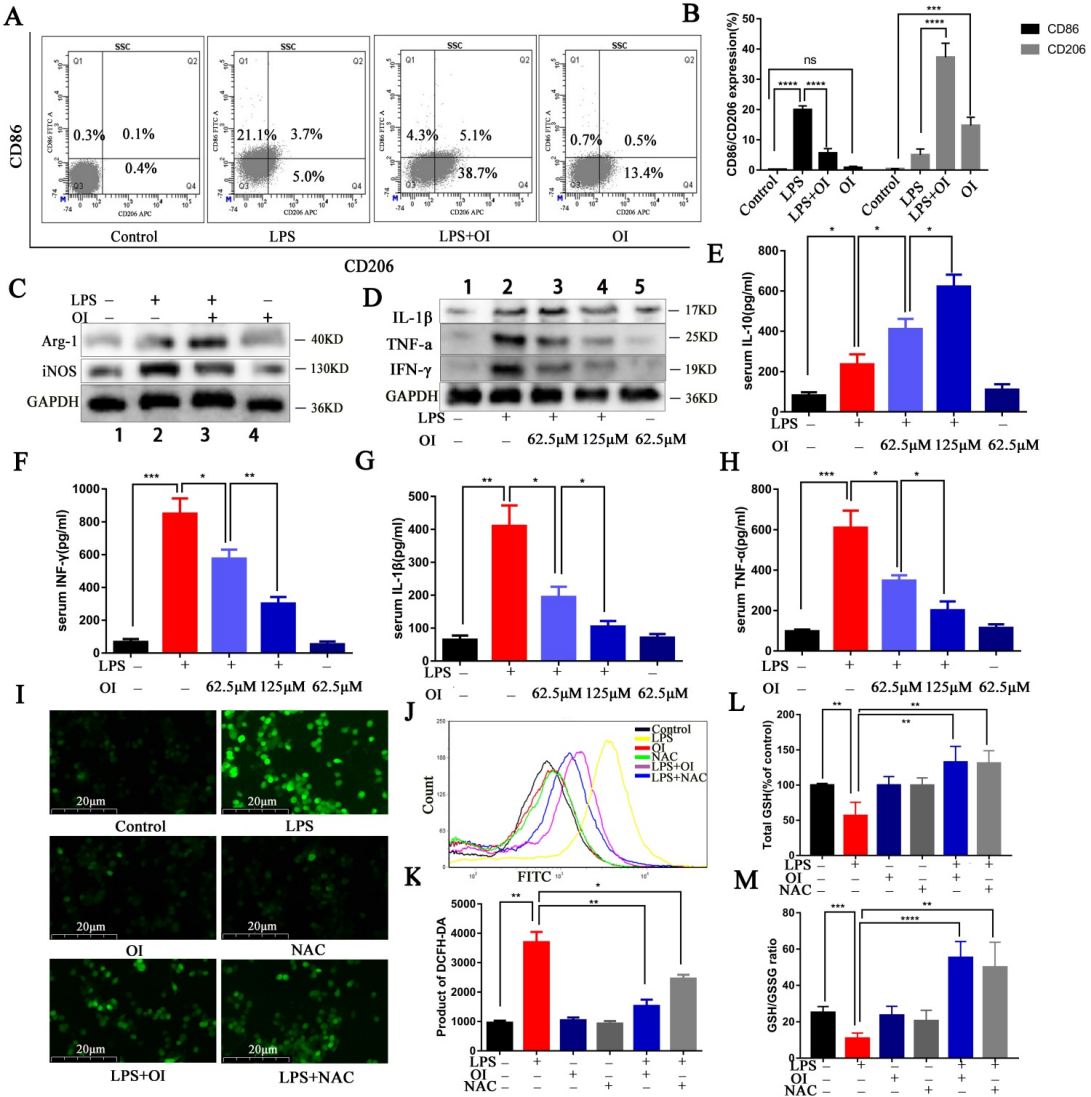
** OI reduced RAW264.7 M1 polarization, increased M2 polarization, and inhibited LPS-induced oxidative stress. (A)** Expression of CD206 and CD86 surface marker proteins in M1 and M2 polarized cells, as measured using flow cytometry. **(B)** Statistical analysis of flow cytometry results. **(C)** Expression of CD206 and CD86 surface marker proteins in M1 and M2 polarized cells, as determined using western blotting. **(D)** Proinflammatory cytokine (IFN-γ, IL-1β, TNF-α) expression in RAW264.7 macrophages, as determined using western blotting. **(E-H)** Pro-inflammatory (IFN-γ, IL-1β, TNF-α) and anti-inflammatory (IL-10) cytokine secretion in RAW264.7 macrophages, as determined using ELISA. **(I-K)** ROS release from RAW264.7 macrophages, as measured using fluorescence microscopy and flow cytometry; magnification, 200×. **(L and M)** GSH levels and the GSH/GSSG in RAW264.7 macrophages. All data are pooled from three independent experiments. Data represent the mean ± SD. ****P < 0.001, ***P < 0.001, **P < 0.01, *P < 0.05.

Macrophages are important inflammatory cells whose polarization determines the balance between pro-inflammatory (M1) and anti-inflammatory (M2) effects. Therefore, we investigated the polarization of the RAW264.7 macrophages using CD86 and i-NOS as M1-type surface markers and CD206 and ARG-1 as M2-type surface markers. We found that CD86 expression significantly increased in RAW264.7 cells treated with LPS, whereas CD206 expression did not. Conversely, CD86 expression was significantly lower in RAW264.7 cells of the LPS + OI group than in those of the LPS group, while CD206 expression was significantly higher. There was no significant difference in CD86 between the OI vs. control group, while there was a significant difference in the increase of CD206 between the OI vs. Control group (Fig. 2A-B). Western blotting revealed that iNOS expression was significantly increased in the LPS group and significantly lower in the LPS + OI group, whereas Arg-1 expression was significantly higher (Fig. 2C).

Although immune overactivation and immune paralysis coexist in the very early phase of sepsis, excessive inflammation is still a hallmark of sepsis. Excessive pro-inflammatory cytokines from monocytes/macrophages are pivotal indices for evaluating the severity of sepsis. We evaluated the effect of OI on the release of RAW264.7 inflammatory cytokines following LPS stimulation. Western blotting confirmed that TNF-α, IL-1β, and IFN-γ expression were significantly higher in RAW264.7 cells treated with LPS and were significantly inhibited by OI (Fig. 2D). ELISA confirmed that no significant change in inflammatory cytokine release was observed in the control group; however, TNF-α, IL-1β, IFN-γ, and IL-10 release were significantly higher in RAW264.7 cells after LPS treatment. Conversely, the release of the pro-inflammatory cytokines TNF-α, IL-1β, and IFN-γ were significantly lower in the LPS + OI group than in the LPS group. In comparison, the release of the anti-inflammatory cytokine IL-10 was significantly higher and positively correlated with OI concentration (Fig. 2E-H).

ROS aggravates inflammation and induces oxidative stress, septic shock, and multi-organ failure. Due to their key role in the inflammatory response, the release of ROS by macrophages causes direct tissue damage; therefore, we examined ROS release by RAW264.7 macrophages. We found that ROS release was significantly increased in RAW264.7 cells from the LPS group and significantly decreased in the LPS+OI group; the inhibitory effect was positively correlated with increasing OI concentration. In addition, the ROS inhibitory effect of OI was similar to that of the ROS-specific inhibitor, NAC (Fig. 2I-K).

Furthermore, LPS treatment significantly decreased total GSH levels and GSH/GSSG ratio in RAW264.7 cells, whereas cells in the LPS+OI group had significantly higher GSH levels and GSH/GSSG ratio. Consistently, the effect of OI on GSH and GSH/GSSG was similar to that of NAC (Fig. 2L-M).

### OI reduces oxidative stress injury in RAW264.7 macrophages by activating the Nrf2 signaling pathway

**Figure 3:**
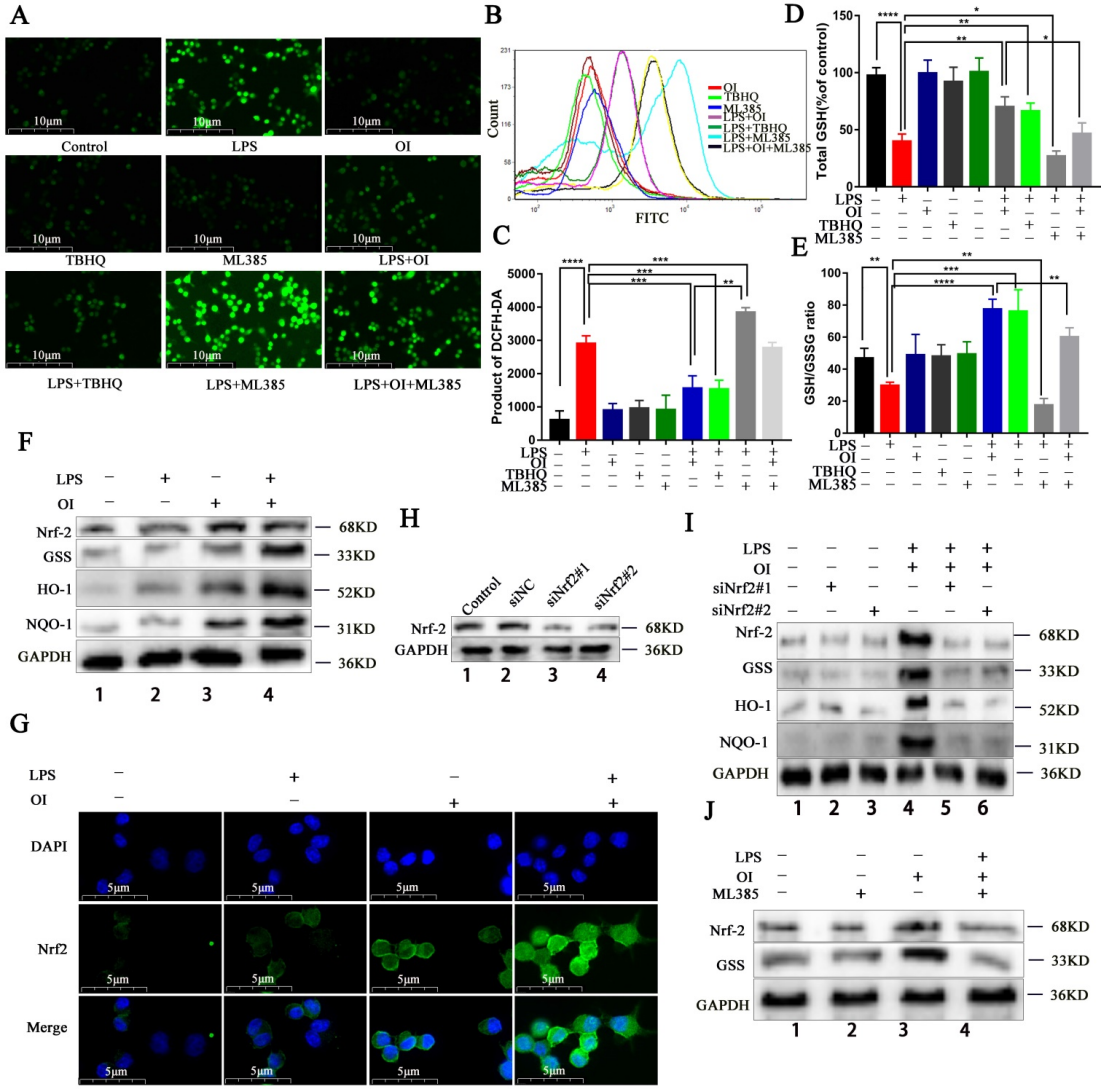
** OI reduces oxidative stress injury in RAW264.7 macrophages by activating the Nrf2 signaling pathway. (A-C)** ROS release from RAW264.7 macrophages, as detected using immunofluorescence and flow cytometry; magnification, 400×. **(D)** GSH synthesis in RAW264.7 macrophages. **(E)** GSH/GSSG ratio in RAW264.7 macrophages. **(F and J)** Nrf2, GSS, HO-1, and NQO-1 protein synthesis, as detected using western blotting. **(G)** Nuclear translocation of Nrf2 in the LPS+OI treatment group, as indicated using immunofluorescence; magnification, 800×. **(H)** Nrf2 protein synthesis after siNrf2#1 and siNrf2#2 knockdown, as detected using western blotting. **(I)** Nrf2, GSS, HO-1, and NQO-1 protein synthesis after siNrf2#1 and siNrf2#2 knockdown and LPS/OI treatment, as detected using western blotting. All data are pooled from three independent experiments. Data represent the mean ± SD. ****P < 0.001, ***P < 0.001, **P < 0.01, *P < 0.05.

Next, we investigated the effect of OI on Nrf2 signaling and oxidative stress injury in RAW264.7 macrophages using the Nrf2-specific agonist TBHQ and the specific inhibitor ML385. OI inhibited LPS-induced ROS release similarly to TBHQ, whereas ML385 enhanced the stimulatory effect of LPS on ROS, and OI partially reversed this inhibitory effect (Fig. 3A-C). Together, these findings confirm that OI reduces oxidative stress in macrophages by activating the Nrf2 signaling pathway to reduce ROS release. In addition, OI inhibited LPS-induced reductions in GSH levels and GSH/GSSG ratio, similarly to TBHQ. GSH and the GSH/GSSG ratio were further reduced in the LPS + ML385 group than in the LPS group, while the reduction in GSH and the GSH/GSSG ratio in the LPS + OI + ML385 group was partially reversed in comparison with in the LPS + OI group (Fig. 3D-E). These results suggest that OI can reduce oxidative stress injury in macrophages by activating the Nrf2 signaling pathway and the production of GSH, which plays a key role in inhibiting oxidative stress.

Previously, Mills *et al.* reported that LPS activates the Nrf2 signaling pathway, while OI stabilizes this effect and significantly increases Nrf2 signaling activation. Herein, we found that the Nrf2 signaling pathway was significantly upregulated in RAW264.7 cells of the LPS + OI group compared to the LPS group, alongside an increase in Nrf2 and GSS protein levels and antioxidant protein (HO-1 and NQO1) synthesis (Fig. 3F). Immunofluorescence also showed that Nrf2 gene transcription and protein expression were significantly higher in the LPS + OI group (Fig. 3G). To better illustrate the oxidation inhibition by OI via the Nrf2 signaling pathway activation, we used the Nrf2 specific inhibitor, ML385. Although Nrf2, GSS, HO-1, and NQO1 expression were elevated in the LPS + OI group, this effect was partially reversed in the LPS + OI + ML385 group (Fig. 3J) and by Nrf2 gene silencing using siRNA (Fig. 3H-I). Therefore, these findings confirm that OI increases GSS synthesis by activating the Nrf2 signaling pathway, thereby inhibiting oxidative stress in macrophages.

### Nrf2 activation by OI is related to PD-L1 expression

To explore the specific mechanism of Nrf2 agonist 4-OI in inhibiting sepsis, RAW264.7 cells were treated in four groups (control, LPS, LPS+OI, and OI). Sequencing was performed 24 hours later, and the sequencing results were analyzed by enrichment analysis.

**Figure 4:**
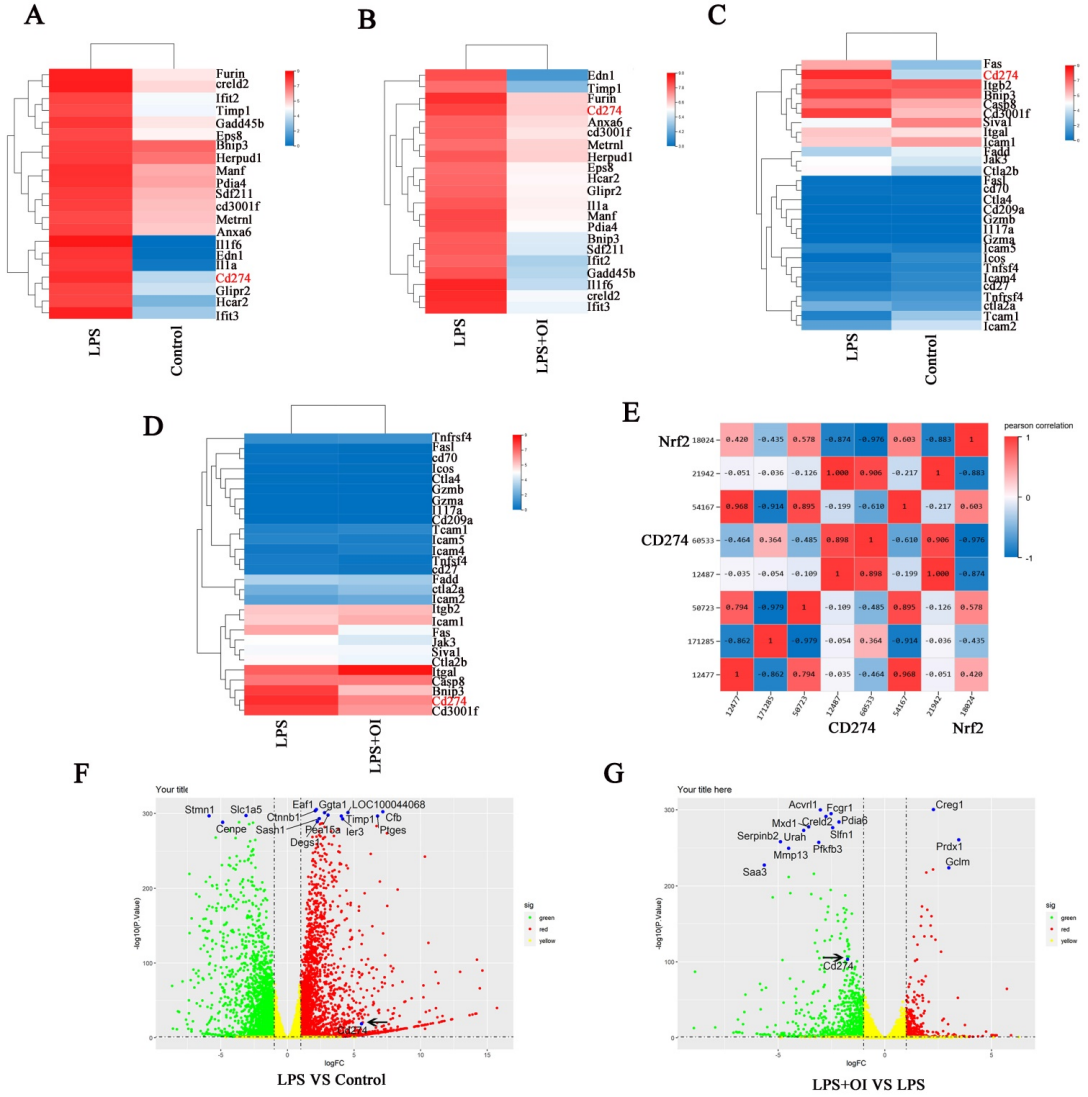
** Nrf2 activator OI affects PD-L1 expression. (A-B)** Genome-wide expression analysis indicated significant enrichment of CD274 expression. **(C-D)** Gene expression enrichment analysis of common immune check points revealed that CD274 expression was significantly enriched. **(E)** RNA-seq analysis revealed a 97.6 % correlation between Nrf2 and CD274. Enrichment analysis of differentially expressed genes indicated that CD274 was significantly upregulated in the LPS group **(F)** and significantly down regulated in the LPS+OI group **(G)**. The black arrow indicates the CD274 gene.

RNA-seq data were used for whole-gene enrichment analysis. The top 21 genes (Furin, creld2, Ifit2, Timp1, Gadd45b, Eps8, Bnip3, Herpud1, Manf, Pdia4, Sdf211, cd3001f, Metrnl, Anxa6, Il1f6, Edn1, Il1a, Cd274, Glipr2, Hcar2, Ifit3) were found in the picture to illustrate the difference in CD274 enrichment analysis between the LPS vs. control group and the LPS vs. LPS + OI group. Genome-wide expression analysis showed that CD274 expression was significantly enriched in the LPS group compared to the LPS + OI group. In contrast, CD274 expression was significantly reduced in the LPS + OI group, with significant differences among each group (Figure 4A-B). Next, we more accurately selected common immune checkpoints in the immune signaling pathway for enrichment analysis and found that CD274 expression was significantly enriched in the LPS group compared to the control group. CD274 expression was significantly reduced in the LPS + OI group compared to the LPS group (Figure 4C-D). Finally, we selected common immune detection points for enrichment analysis and found that Nrf2 and CD274 displayed a significant correlation of 97.6% (Fig. 4E).

We conducted enrichment analysis on the differentially expressed genes in each group. CD274 was significantly upregulated in the LPS group compared to the control group and significantly downregulated in the LPS + OI group, suggesting that OI could regulate CD274 expression via Nrf2 (Fig. 4F-G).

According to the sequencing results, we found that 4-OI significantly regulated the expression of CD274 gene and negatively regulated the expression of CD274 gene. As an Nrf2 agonist, does 4-OI show that Nrf2 has a strong regulatory effect on the expression of CD274 gene? In the following experiments, we will explore the specific molecular mechanism of Nrf2 gene regulating CD274 gene in detail.

### OI inhibits PD-L1 activation by LPS, while PD-L1 expression is not controlled by ROS

**Figure 5:**
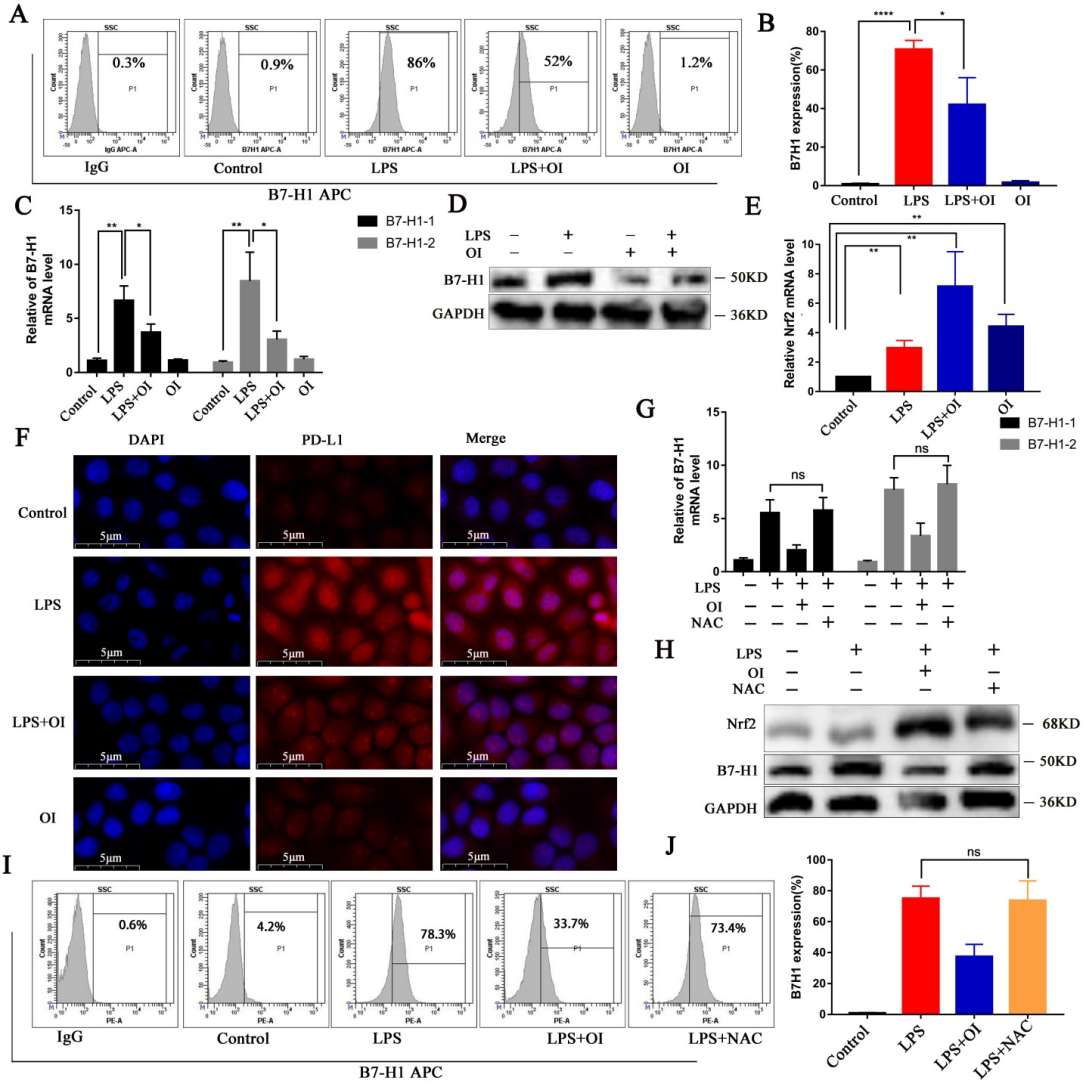
** OI inhibits B7-H1 activation by LPS, but B7-H1 expression is not controlled by redox. (A and B)** B7-H1 protein synthesis in RAW264.7 macrophages, as detected using flow cytometry and statistical analysis. **(C)** B7-H1 expression in RAW264.7 macrophages, as determined using RT-PCR. **(E)** Nrf2 expression in RAW264.7 macrophages, as determined using RT-PCR. **(D and F)** B7-H1 protein synthesis, as detected using western blotting and immunofluorescence; magnification, 800×. **(G)** B7-H1 expression in RAW264.7 macrophages after treatment with the ROS-specific inhibitor NAC, as measured using RT-PCR. **(H)** B7-H1 protein synthesis in RAW264.7 macrophages after treatment with the ROS-specific inhibitor, NAC, as determined using western blotting. **(I and J)** B7-H1 protein synthesis in RAW264.7 macrophages after treatment with NAC, as detected using flow cytometry and statistical analysis. All data are pooled from three independent experiments. Data represent the mean ± SD. ****P < 0.001, ***P < 0.001, **P < 0.01, *P < 0.05, NS, not significant.

To determine the effect of OI on PD-L1 (B7-H1) protein synthesis, we measured the expression of RAW264.7 cell surface proteins using flow cytometry. B7-H1 synthesis was significantly higher in the LPS group than in the control group but was inhibited in the LPS + OI group (Fig. 5A-B). In addition, qRT-PCR verified that B7-H1 expression was significantly higher in the LPS group than in the control group and was significantly lower in the LPS + OI group than in the LPS group (Fig. 5C). WB confirmed that B7-H1 synthesis was significantly higher in the LPS group than in the control group but was inhibited in the LPS + OI group (Fig. 5D), and immunofluorescence proved the above (Fig. 5F). Conversely, Nrf2 gene expression was lower in the LPS and OI groups. However, it was significantly increased in the LPS + OI group (Fig. 5E).

Since Nrf2 regulates the expression of several genes involved in ROS control, we investigated whether Nrf2-mediated PD-L1 gene induction and inhibition depend on ROS. LPS significantly upregulated ROS levels, while NAC, a precursor of GSH, reduced ROS levels to the basic level. However, NAC did not affect LPS-induced B7-H1 gene transcription (Fig. 5G) or protein expression (Fig. 5H-J). Therefore, reducing ROS levels does not induce PD-L1 gene expression.

### Nrf2 signaling can inhibit PD-L1 gene expression and protein synthesis

**Figure 6:**
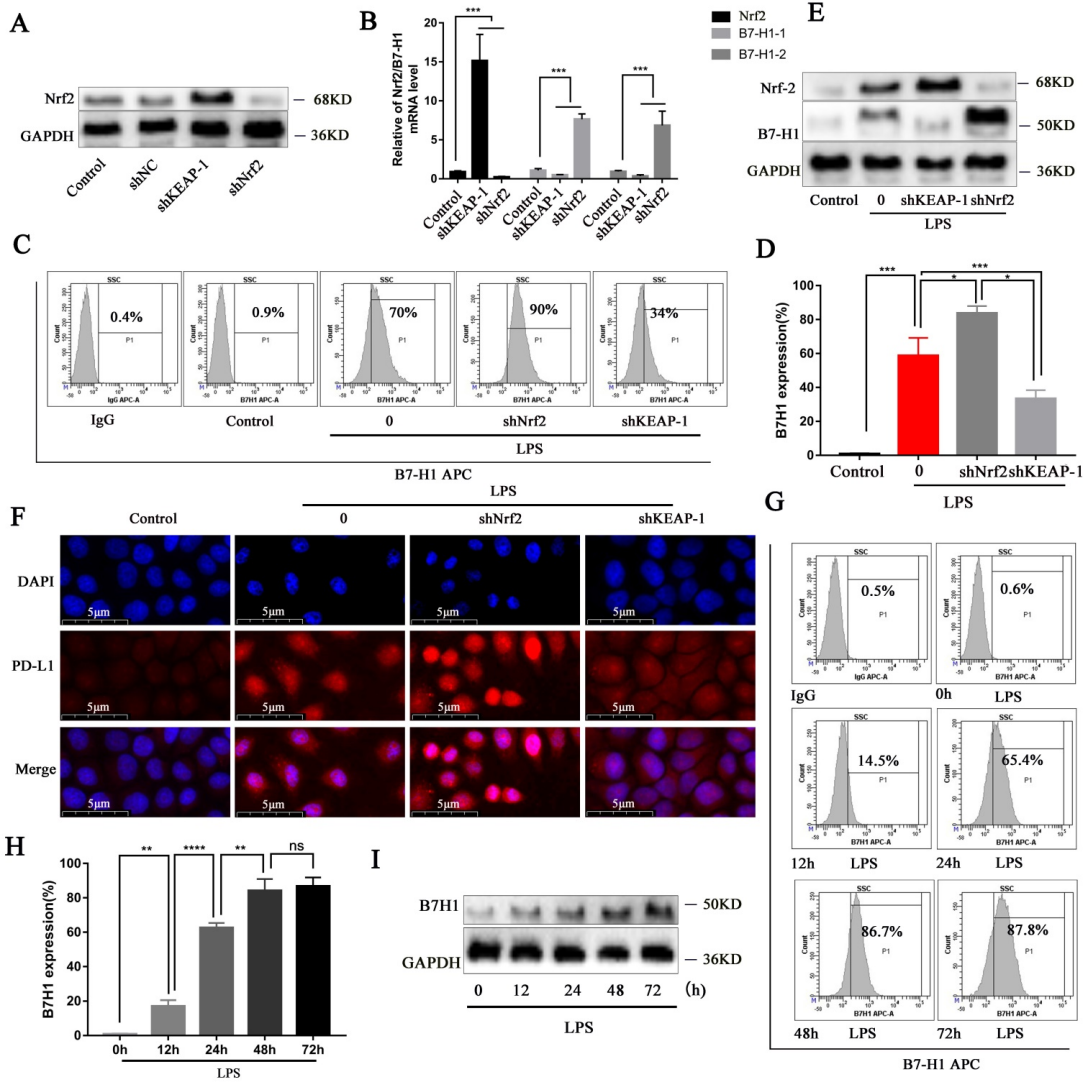
** Nrf2 signaling pathway can inhibit B7-H1 gene expression and protein synthesis, while LPS promotes B7-H1 protein expression up to 48 h. (A)** Nrf2 protein synthesis after Nrf2 knockdown and overexpression (shKEAP-1), as detected using western blotting. **(B)** Nrf2 and B7-H1 gene expression after Nrf2 knockdown (shNrf2) and overexpression (shKEAP-1), as detected using RT-PCR. **(C and D)** B7-H1 protein synthesis in RAW264.7 macrophages, as measured using flow cytometry and statistical analysis. **(E and F)** Nrf2 and B7-H1 protein synthesis detected using western blotting and immunofluorescence; magnification, 800×. **(G and H)** B7-H1 protein synthesis in RAW264.7 macrophages treated with LPS at different time points, as detected using flow cytometry and statistical analysis. **(I)** B7-H1 protein synthesis at different time points, as detected using western blotting. All data are pooled from three independent experiments. Data represent the mean ± SD. ****P < 0.001, ***P < 0.001, **P < 0.01, *P < 0.05; NS, not significant.

Since we confirmed that OI activates the Nrf2 signaling pathway and thereby inhibiting B7-H1 expression and synthesis, we evaluated the B7-H1 gene and protein synthesis following Nrf2 gene lentiviral knockout and overexpression, whose efficiency was confirmed (Fig. 6A). Nrf2 gene overexpression significantly decreased B7-H1 gene expression, whereas Nrf2 gene knockout significantly increased B7-H1 expression (Fig. 6B). Flow cytometry confirmed that in LPS-treated RAW264.7 cells, B7-H1 synthesis was higher following Nrf2 gene knockout but lower when Nrf2 was overexpressed (shKeap1; Fig. 6C-D), consistent with the immunoblotting and immunofluorescence results (Fig. 6E-F). Moreover, flow cytometry showed that B7-H1 synthesis was significantly increased in RAW264.7 cells from 0-48 h and 48-72 h (Fig. 6G-H), consistent with the immunoblot findings (Fig. 6I).

### Nrf2 can specifically bind to the PD-L1 gene and negatively regulate PD-L1

**Figure 7:**
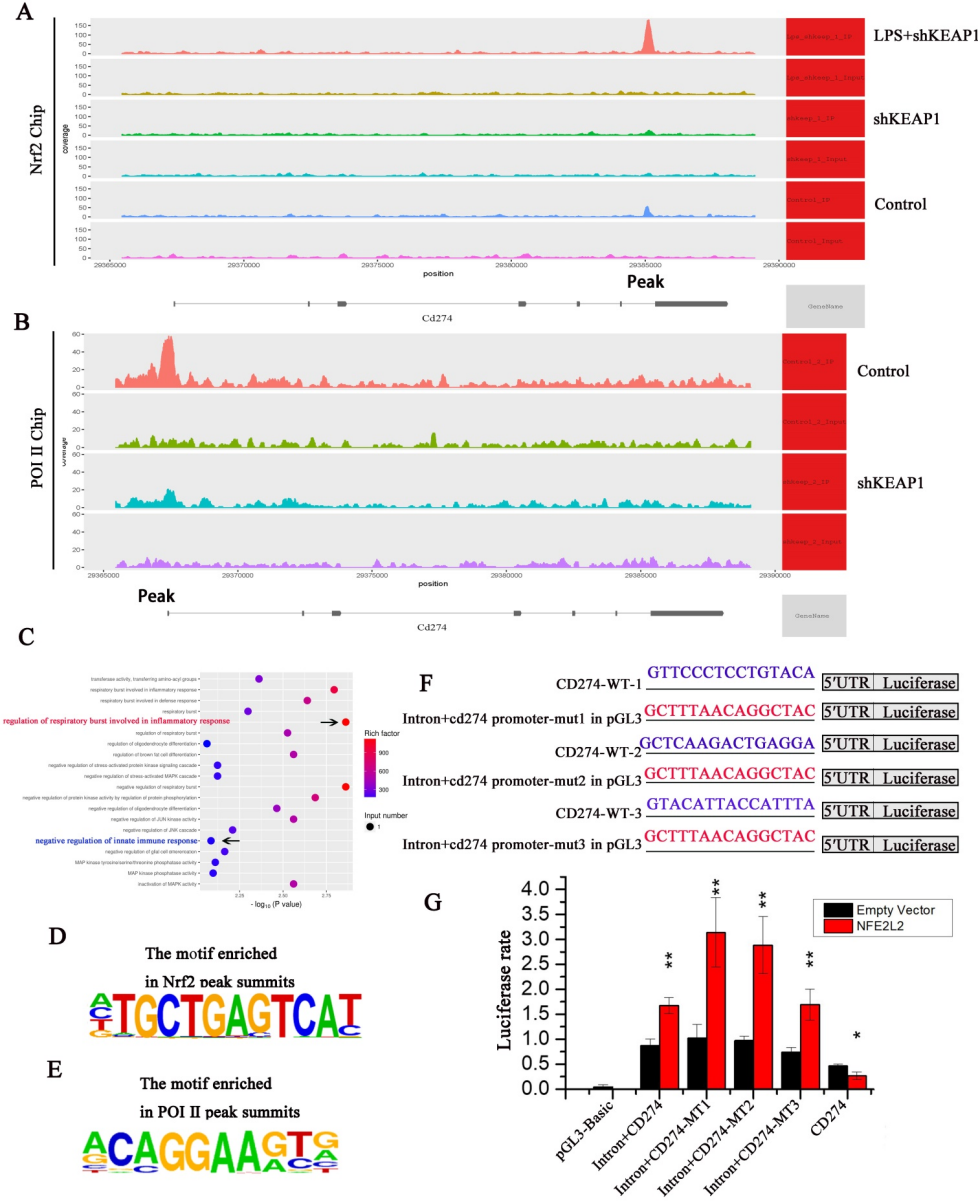
** Nrf2 can specifically bind to the B7-H1 gene and negative regulate its transcription. (A)** Intron-specific binding peaks between Nrf2 and the CD274 gene. **(B)** Intron-specific binding peaks between Pol II and the CD274 gene. **(C)** KEGG-pathway enrichment analysis revealed a significant correlation with the "regulation of respiratory burst involvement in inflammatory response" and "negative regulation of innate immune response". **(D)** Intron-specific binding regions in Nrf2 and CD274 predicted using Motif. **(E)** Intron-specific binding regions in Pol II and CD274 predicted using Motif. **(F)** Vector luciferase reporter pattern showing three possible intron binding site mutations were obtained from the wild-type (WT). **(G)** Increased luciferase expression in the reporter vector relative to the intra gene + CD274-MT1, intra gene + CD274-MT2, and intra gene + CD274-MT3 mutations.

To determine whether Nrf2 binds directly to the PD-L1 (CD274) gene, we conducted an Nrf2 ChIP-seq analysis. Specific binding to Nrf2 was observed in the CD274 intron region; however, the binding peak between Nrf2 and CD274 significantly changed under different conditions. When Nrf2 was overexpressed, the binding peak decreased compared to the control group but significantly increased in cells treated with LPS (Fig. 7A), confirming that Nrf2 inhibits CD274 gene expression and protein synthesis. Sequencing analysis of ChIP products revealed that Nrf2 gene had three binding sites with intron 6 of CD274 gene, which were: Base 935-950 GTTCCCTCCTGTACA, base 945-960 GTACATTACCATTTA, base 995-1011 GCTCAAGACTGAGGA.

Through ChIP-seq analysis, we identified a motif with a high binding probability (Fig. 7D) and analyzed three possible binding sites for Nrf2 in CD274, namely CD274-MT1, CD274-MT2, and CD274-MT3. We then mutated these binding sites (Fig. 7F) and conducted luciferase reporter gene assays. Compared to the negative control, luciferase expression was significantly increased in the CD274 promoter reporter vector of cloned mice, indicating that the clonal regions had promoter activity. In addition, Nrf2 significantly improved the activity of wild-type and mutant intronic promoters, indicating that the intron sequence may act as an enhancer. When intron + CD274-MT1, intron + CD274-MT2, and intron + CD274-MT3 were mutated, relative luciferase expression was increased, suggesting that Nrf2 may inhibit the CD274 promoter via these three sites (Fig. 7G).

Next, we performed ChIP-seq experiments with Pol II, the key enzyme in gene transcription. Peak CD274 binding sites were lower in the shKeap1 group, suggesting that Nrf2 gene expression reduces Pol II binding and significantly reduces CD274 transcription (Fig. 7B). Through ChIP-seq analysis, we identified a motif with a high binding probability (Fig. 7E) and analyzed possible binding sites for Pol II in CD274. KEGG-pathway enrichment analysis of the Nrf2 ChIP-seq results showed a significant correlation between “regulation of respiratory burst involved in inflammatory response” and “negative regulation of innate immune response” (Fig. 7C). This suggests that Nrf2 may inhibit the B7-H1 gene transcription.

### PD-L1 antibody treatment reduced mortality in the mouse CLP model of sepsis

**Figure 8:**
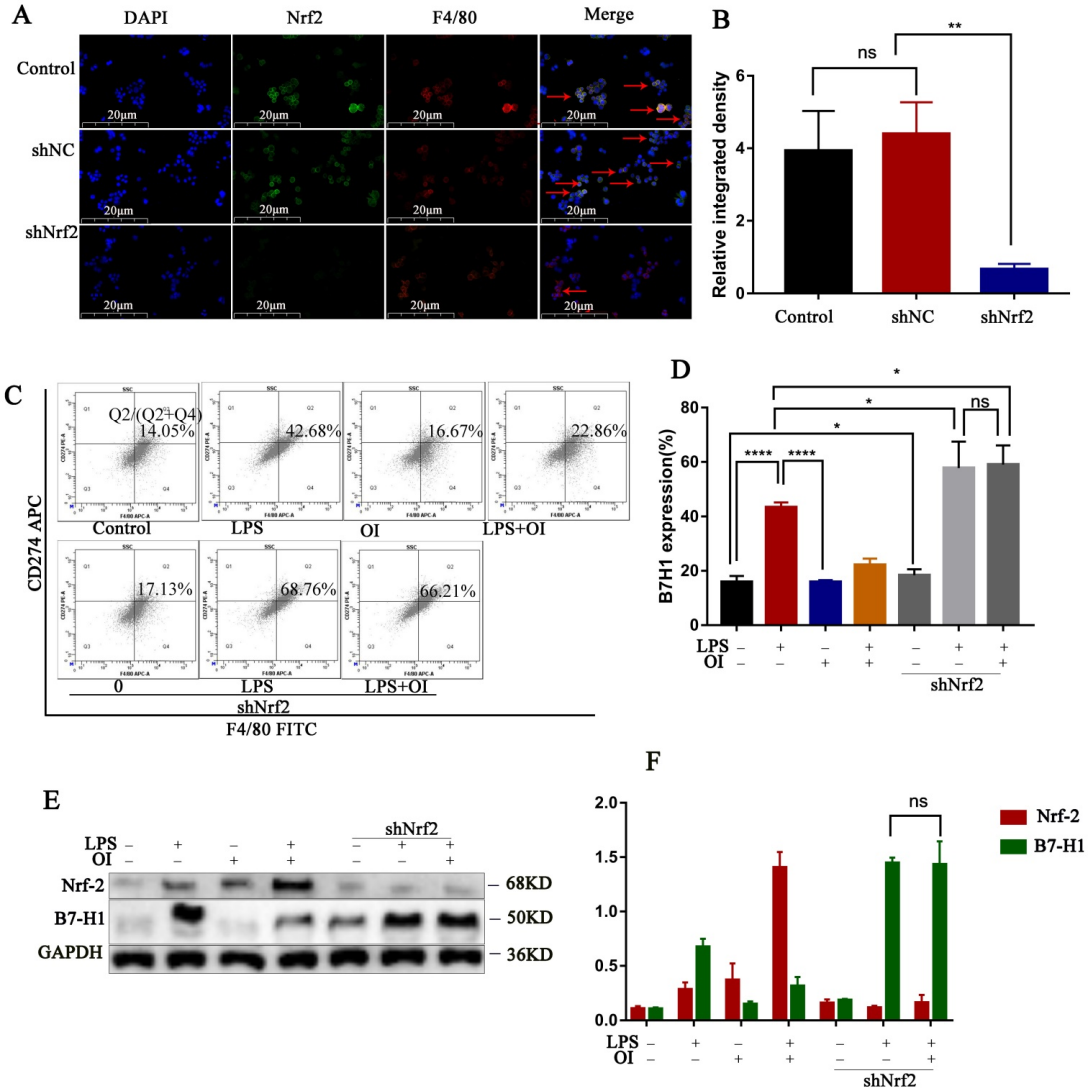
***In vivo* experiments confirmed that Nrf2 gene inhibited B7-H1 protein synthesis. (A and B)** Nrf2 expression in extracted mouse peritoneal macrophages after immunofluorescence double staining (F4/80 and Nrf2). The red arrows indicate double - labeled positive cells; magnification, 200×. **(C and D) B7-H1** expression in mouse peritoneal macrophages, as determined using flow cytometry and statistical analysis. **(E and F)** Nrf2 and **B7-H1** protein synthesis in mouse peritoneal macrophages, as measured using western blotting. Data represent the mean ± SD. ****P < 0.001, ***P < 0.001, **P < 0.01, *P < 0.05; NS, not significant.

**Figure 9:**
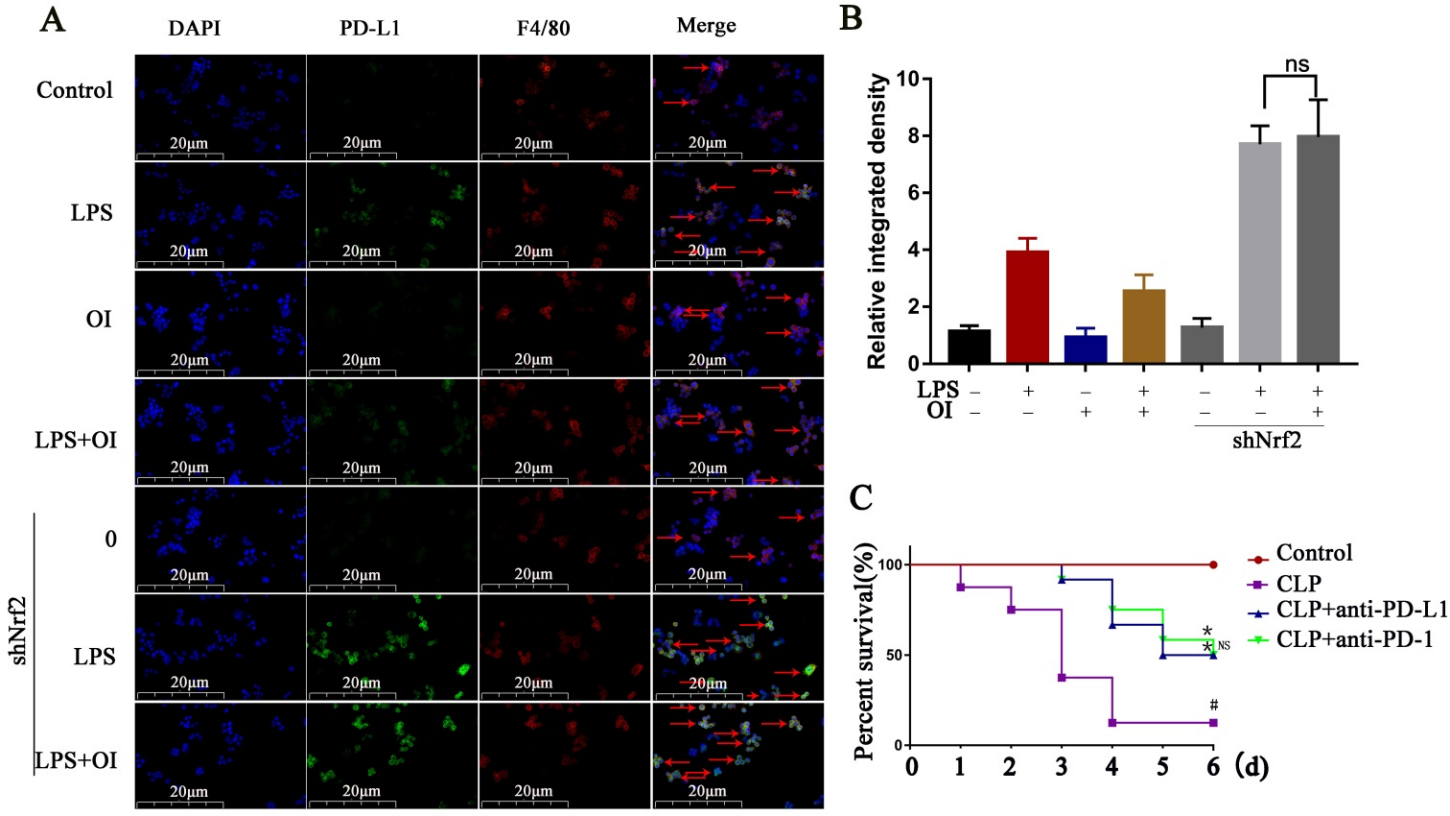
** PD-L1 antibody treatment reduced mortality in the mouse CLP model of sepsis. (A and B)** PD-L1 expression in extracted mouse peritoneal macrophages after immunofluorescence double staining (F4/80 and PD-L1). The red arrows indicate double - labeled positive cells. Magnification, 200×. **(C)** Anti-PD-L1 reduces mortality in mice with sepsis (n=12) #*P* < 0.05 vs. control, *P < 0.05 vs CLP. No statistical significance was observed between the CLP+anti-PD-L1 and CLP+anti-PD-L1 groups. All data are pooled from two independent experiments. Data represent the mean ± SD. ****P < 0.001, ***P < 0.001, **P < 0.01, *P < 0.05; NS, not significant.

Nrf2 gene of mouse peritoneal macrophages was knocked down by intraperitoneal injection of lentiviral shRNA. To confirm our conclusion, we applied LPS induction to the extracted macrophages. Immunofluorescence double staining with F4/80 and Nrf2 indicated that the Nrf2 protein expression was significantly reduced in macrophages in the siNrf2 group (Fig. 8A). Statistical analysis of immunofluorescence showed that the expression of Nrf2 protein in peritoneal macrophages was significantly decreased after lentiviral shRNA knockdown (Fig. 8B), while flow cytometry showed that LPS significantly increased PD-L1 expression in macrophages from the control and shNrf2 groups, while OI significantly inhibited this effect. However, the stimulatory effect of LPS on PD-L1 was not prevented by OI when the Nrf2 gene was knocked down (Fig. 8C). Statistical analysis showed that there was no difference in B7-H1 protein expression between shNrf2+LPS group and shNrf2+LPS+OI group (Fig. 8D), as confirmed using western blotting (Fig. 8E-F). The same conclusion was obtained by immunofluorescence assay (Fig. 9A-B).

Finally, we investigated the effect of inhibiting PD-L1 (B7-H1) expression on mice mortality. The control group had a survival rate of 100% over the observation period (1-6 d), whereas the survival rates of mice in the CLP group were 91.6, 83.3, 33.3, and 8.3% after 1, 2, 3, and 4-6 d, respectively. In the CLP + anti-PD-1 (200 ng/kg) group, the survival rates were 100% at 0-2 d, 91.6% at 3 d, 83.3% at 4 d, 66.7% at 5 d, and 50% at 6 d, whereas those in the CLP + anti-PD-L1 (200 ng/kg) group were 100% at 0-2 d, 91.6% at 3 d, 75.0% at 4 d, and 50% at 5-6 d. Together, these findings confirm that anti-PD-L1 could significantly reduce the mortality of mice with sepsis (Fig. 9C).

## Discussion

The physiological and pathological characteristics of sepsis are associated with an imbalance in the immune system and an increased inflammatory response during the early stage of the disease. The early symptoms of sepsis are exacerbated by other diseases, including diabetes, hypertension, or heart disease, and are influenced by the types of pathogenic bacteria and their ability to release toxins. However, the main reason for the early deterioration in patients with sepsis is the inflammatory cytokine storms caused by the release of pro-inflammatory cytokines and subsequent ROS production, which causes oxidative stress damage. However, progressive sepsis often leads to a suppressed immune response which significantly increases the risk of secondary infections and, ultimately, the fatality rate 6, 58-60.

Various treatments and auxiliary drugs, such as corticosteroids, insulin, and recombinant activated protein C have been used to treat sepsis. However, these treatments can induce potentially life-threatening side effects, such as cardiovascular disease and infection, sleep disorders, and stomach discomfort 59, 61, 62. Itaconate is an anti-inflammatory metabolite that has been shown to exert anti-inflammatory effects in systemic lupus erythematosus and liver ischemia/reperfusion injury by alkylating Keap1 and activating the Nrf2 signaling pathway to inhibit oxidative stress injury 56, 63-65. In this study, we confirmed that OI, an important itaconate derivative, can significantly reduce the mortality of mice with sepsis by reducing tissue and organ damage, reducing the expression of pro-inflammatory cytokines in the blood, and increasing the expression of anti-inflammatory cytokines.

An important aspect of the early pathogenesis of sepsis is the production of large amounts of ROS, which leads to excessive oxidation 66, 67. Most oxygen free radicals in sepsis are derived from phagocytes, particularly monocytes/macrophages, which synthesize and secrete numerous pro-inflammatory cytokines when stimulated by pathogens, causing a respiratory burst and the release of ROS, which leads to oxidative stress 34, 68, 69. In this study, we found that OI can significantly reduce ROS release in RAW264.7 cells treated with LPS and resist decreases in GSH levels and the GSH/GSSG ratio, which suggests that endogenous metabolite OI may modulate macrophage function.

The inflammatory imbalance caused by sepsis leads to persistent immune cell dysfunction, predisposing patients to secondary infections and enhancing disease progression. Therefore, advanced sepsis immunosuppression can be an important cause of uncontrolled infection, organ dysfunction or failure, and even death 10, 15, 21. PD-1 is an important immunosuppressive molecule belonging to the B7 family whose combination with PD-L1 leads to the tyrosine phosphorylation of ITIM or ITSM functional domains, the dephosphorylation of signal transduction components downstream of the TCR/CD28 pathway, which inhibits the signal transduction in this pathway, thereby weakening the activation effect of APC in T cells. Therefore, the molecular co-inhibition of PD-1/PD-L1 plays an important role in immunosuppression in patients with sepsis 12, 14, 22.

Herein, we found that PD-1 and PD-L1 expression increased significantly during inflammation in adaptive immune cells such as T cells and innate immune cells (macrophages, neutrophils, and monocytes), leading to suppressed immune cell function and subsequent immune dysfunction in tissue and organs 70-72. Consistently, numerous studies have shown that the PD-1/PD-L1 pathway plays an important role in promoting the progression of sepsis. For instance, the systemic inflammatory response was reported to be significantly lower in PD-1-/- mice with sepsis than in wild-type mice, as was the mortality 72. In addition, when the peritoneal macrophage function of wild-type mice is absent or reduced, its phagocytosis function is reduced, and its ability to remove pathogenic microorganisms is reduced, thus increasing the mortality rate 72. Consistently, we confirmed that treatment with PD-L1 antibodies significantly increased the survival rate of mice with sepsis, suggesting that blocking the PD-1/PD-L1 pathway exerts a protective effect in mice with sepsis.

Monocytes/macrophages are an important part of the innate immune response to pathogens; however, innate immune cells can directly lead to immunosuppression in sepsis and accelerate lymphocyte apoptosis, leading to an exacerbated immune response. Huang *et al.* found that PD-L1 gene and protein expressions were increased in mice 12 h pose-surgery and continued to increase for 24 to 48 h with similar subsequent changes observed in their blood and liver macrophages 71-74. We also found that PD-L1 expression increased in RAW264.7 macrophages 0-12 h following LPS stimulation, peaking over 12-48 h and then slowing after 48 h. Consistently, Guignant et al. demonstrated significant increases in monocyte/macrophage PD-L1 expression for 3-5 d in the blood of patients with severe sepsis; however, the mechanism underlying PD-L1 expression in macrophages remains unclear 75.

In this study, we aimed to elucidate the molecular basis of the anti-inflammatory effects of Nrf2 and found that Nrf2 regulates intracellular ROS and inhibits PD-L1 gene expression at the transcriptional level. We believe that this expands our understanding of the inflammation-related functions of Nrf2 since it has been previously believed that Nrf2 only controls oxidative stress and exerts anti-inflammatory effects via ROS elimination. Although Nrf2 is a transcription factor, we found that Nrf2 can also act as a transcriptional suppressor by directly binding to DNA in the PD-L1 gene and inhibiting transcription in a ROS-independent manner. In particular, we found that Nrf2 inhibits the recruitment of Pol II to the transcriptional start site of the PD-L1 gene, thus inhibiting its transcription. Interestingly, although we found that Nrf2 inhibits PD-L1 transcription in macrophages, previous studies have found that Nrf2 can act as a transcriptional activator. Notably, our luciferase reporter assays and Chip-SEQ analysis indicated that Nrf2 inhibits PD-L1 transcription at different gene binding sites, suggesting that Nrf2 interferes with the transcriptional activation system for inflammatory responses. Although the complex molecular basis underlying Nrf2-induced transcriptional inhibition warrants further investigations, our results suggest that Nrf2 negatively regulates the immunosuppressive target gene, PD-L1.

We further investigated the mechanism via which Nrf2 inhibits PD-L1 transcription by knocking out Nrf2 in RAW264.7 macrophages and mouse peritoneal macrophages, using a mouse model of LPS-induced sepsis. LPS significantly increased PD-L1 expression in abdominal macrophages, whereas the Nrf2 activator, OI, inhibited this effect. Moreover, Nrf2 knockdown promoted the stimulatory effect of LPS on PD-LI and reduced the inhibitory effect of OI. Therefore, these findings confirm that Nrf2 acts as a transcriptional inhibitor of PD-L1 expression and confirm that OI activates Nrf2 to inhibit PD-L1 expression in septic macrophages. Together, our findings may explain the mechanism underlying the efficacy of OI as a potential treatment option for sepsis.

In this study, we confirmed that OI significantly reduces the release of inflammatory factors and oxidative stress injury during the early stage of sepsis, thereby protecting tissue and organ function. In the advanced and later stages, OI can regulate immune function and prevent immune suppression by inhibiting the expression of PD-L1, an important immune detection point. Although we verified that Nrf2 inhibits the recruitment of Pol II to the PD-L1 transcriptional start site, thus inhibiting its transcription, we also found that Nrf2 can specifically bind to PD-L1 DNA and inhibit its transcription as an immunosuppressive factor. In contrast to the conclusions drawn by the authors in the present manuscript, several publications show that Nrf2 is a positive transcriptional regulator of PD-L1, such as lung cancer and melanoma 76, 77. Best *et al.* identified an immunosuppressive microenvironment in Keap1f/f/Ptenf/f tumor-bearing lungs, correlating with high levels of PD-L1 expression, a characteristic that could be exploited therapeutically. Indeed, anti-PD-1/anti-CTLA-4 treatment of Keap1f/f/Ptenf/f spontaneous lung tumors resulted in tumor regression. These data provide important insights into the Keap1/Nrf2 pathway under homeostatic and oncogenic conditions in the lung. They demonstrated that Keap1/Nrf2 pathway promotes PD-L1 expression in the microenvironment from the perspective of metabolism and tumor immune microenvironment, thus providing a new target for lung cancer immunotherapy. Zhu *et al.* found a potential NRF2-binding site (NBE) in the first intron of the PD-L1 gene to promote the transcription of CD274. The reduction of NRF2 can significantly induce CD8+ and CD4+ T cells to infiltrate tumors and inhibit the melanoma progression. Inhibition of NRF2 combined with anti-PD-1 therapy can enhance its antitumor function. We think the reason for this difference may be the nature of the disease, the characteristics of the cell and the different binding sites of the transcription factors. Notably, Nrf2 deficiency has been found to cause spontaneous autoimmune phenotypes, further supporting the possibility that Nrf2 may act as an inducer in other autoimmune diseases. Our study has established that Nrf2 induction could be an alternative target for anti-inflammatory drugs. In addition, we elucidated the specific molecular mechanism underlying the transcriptional regulation of PD-L1 by Nrf2, thus providing potential targets for immunotherapies against tumors, inflammation, and other associated diseases.

## Figures and Tables

**Figure 10:**
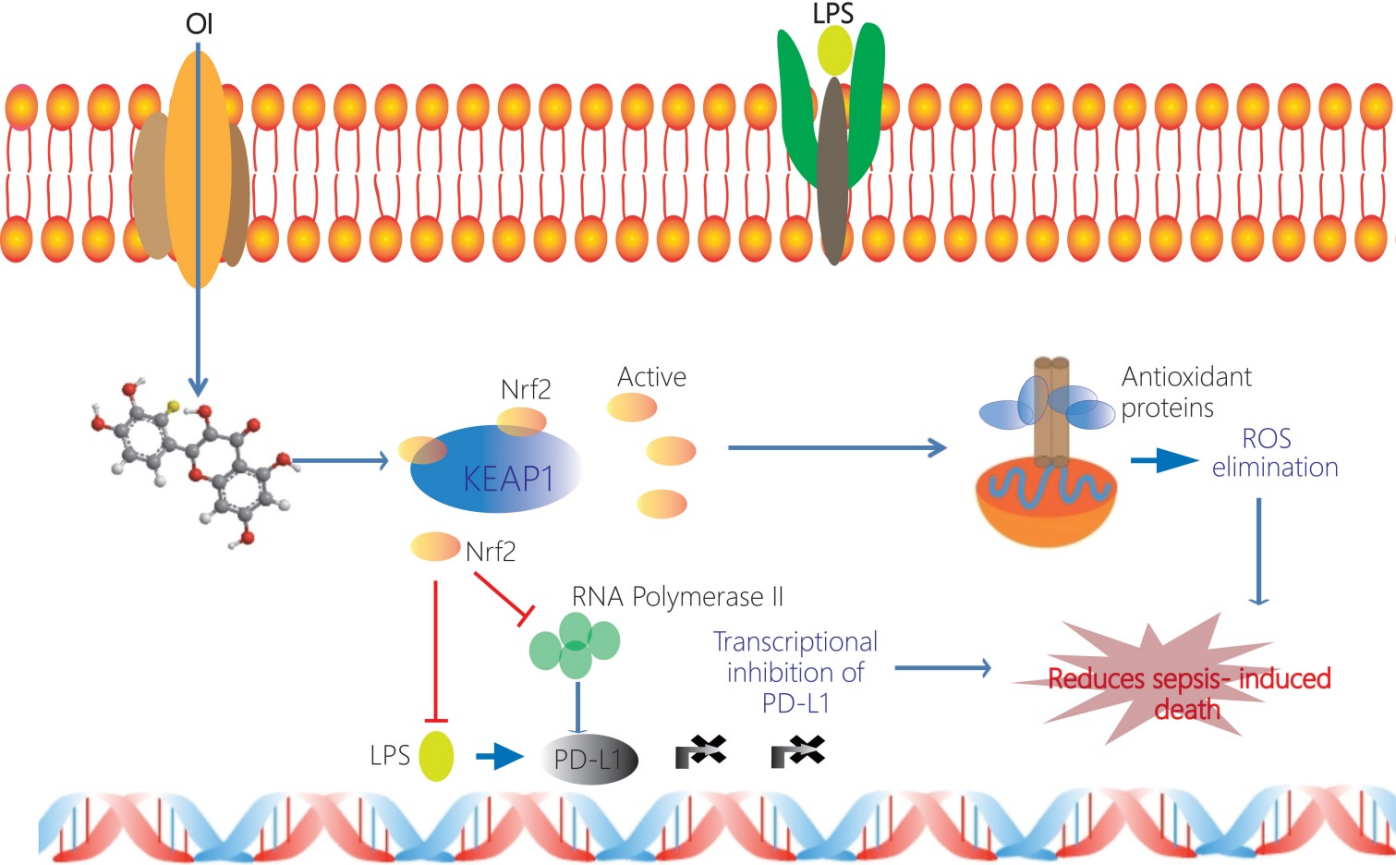
** Schematic representation of the itaconate-mediated anti-inflammatory effect. Itaconate promotes the dissociation of KEAP1 from Nrf2, thus activating Nrf2 gene.** Nrf2 binds to the PD-L1 genes and inhibits their transcription. Undeniably, Nrf2 inhibits the transcription of RNA polymerase 2 and thus indirectly inhibits the transcription of PD-L1. At the same time Nrf2 upregulates expression of genes coding antioxidant proteins. These antioxidant proteins eliminate ROS and subsequently contribute to the anti-inflammation. Itaconate may directly decrease the expression of proinflammatory cytokines and promote the expression of anti-inflammatory cytokines.
